# The role of carbohydrates in canine and feline nutrition

**DOI:** 10.1093/af/vfae017

**Published:** 2024-06-20

**Authors:** Emanuela Kayser, Shannon E Finet, Maria R C de Godoy

**Affiliations:** Division of Nutritional Sciences, University of Illinois at Urbana-Champaign, Urbana, IL, USA; Department of Animal Sciences, University of Illinois at Urbana-Champaign, Urbana, IL, USA; Division of Nutritional Sciences, University of Illinois at Urbana-Champaign, Urbana, IL, USA; Department of Animal Sciences, University of Illinois at Urbana-Champaign, Urbana, IL, USA

**Keywords:** carbohydrate, cat, dog, dietary fiber, gut health, microbiota

**Implications**

- Carbohydrates are heterogenous compounds with diverse properties.
- Dogs and cats do not have a nutritional requirement for carbohydrates.
- Carbohydrates are functional ingredients for pet food processing.
- Starch is an important dietary source of glucose.
- Dietary fibers have a variety of physiological properties, aid in gut health and modulation of microbiota.

## Introduction

Companion animals play an essential role in people’s lives and are now considered to be and treated as family members in most households worldwide, behavior which has been named the ‘interspecies family’ phenomenon (Owens and Grauerholz, 2019). Therefore, human and pet food trends have been converging for years, leading to an increased importance placed on pet health and longevity reflected in the pet food market. Among the nutrient categories, carbohydrates (CHO) have gained renewed interest in the pet food industry, as they have important roles in energy metabolism, modulation of bowel movement, immune function, and gut microbiota profile (Thompson, 2008).

## Carbohydrates Definition

**Figure 1.**
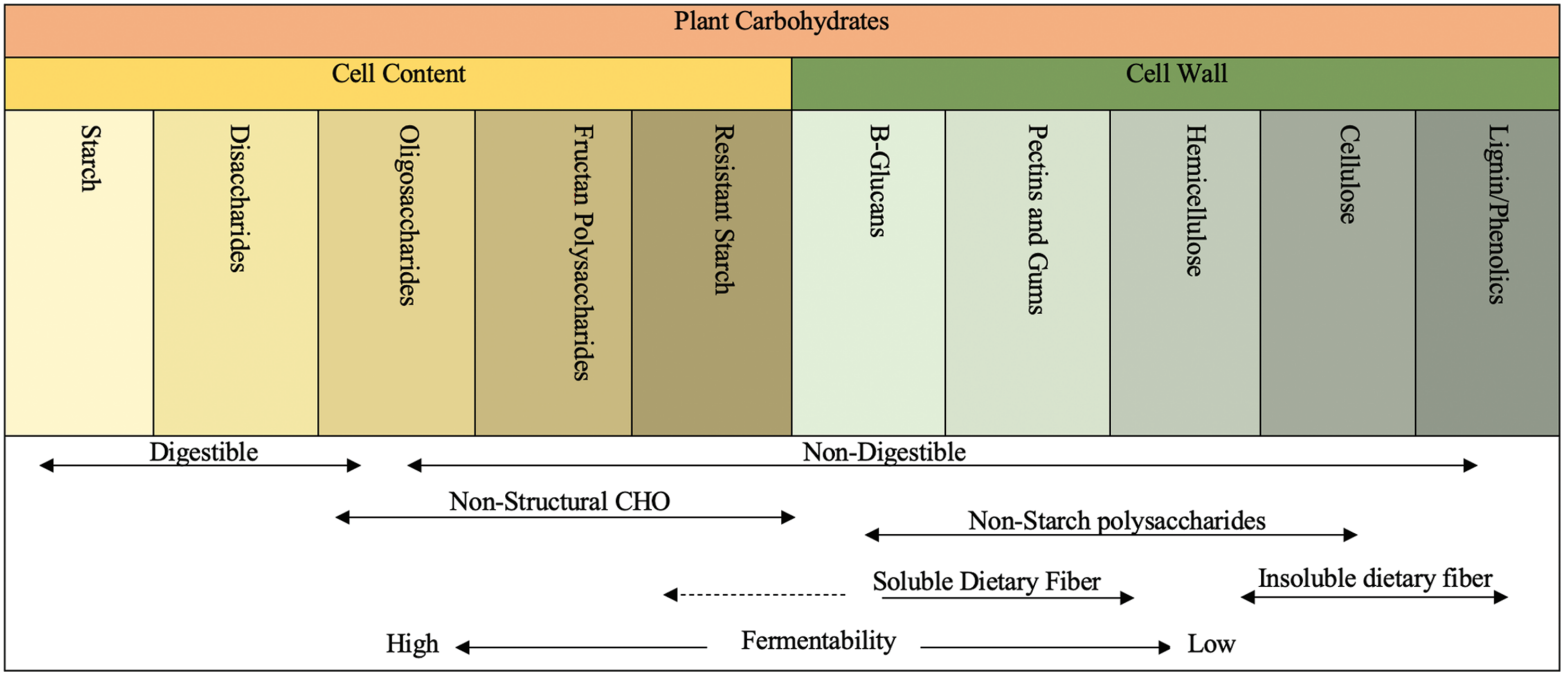
Classification and different categories of dietary carbohydrates.* *Adapted from NRC (2012).

As for other macronutrients, the primary classification of dietary CHO, as proposed at the Joint Food and Agriculture Organization (FAO)/World Health organization (WHO), is by molecular size, as determined by degree of polymerization (DP), the type of linkage (α or β), and character of individual monomers (FAO, 1998). The three major CHO groups are divided based on DP: monosaccharides (DP 1 to 2), oligosaccharides (DP 3 to 9), and polysaccharides (DP ⩾ 10) (Cummings and Stephen, 2007). Carbohydrates can be found in plant cell contents and walls, those differ in their chemical structure and properties, which does not allow a simple translation into nutritional effects (Kaushik et al., 2022). CHO can also be classified according to the degree of their digestion, absorption or fermentation in the upper or lower digestive tract (Adebowale et al., 2019; Figure 1).

**Figure 2.**
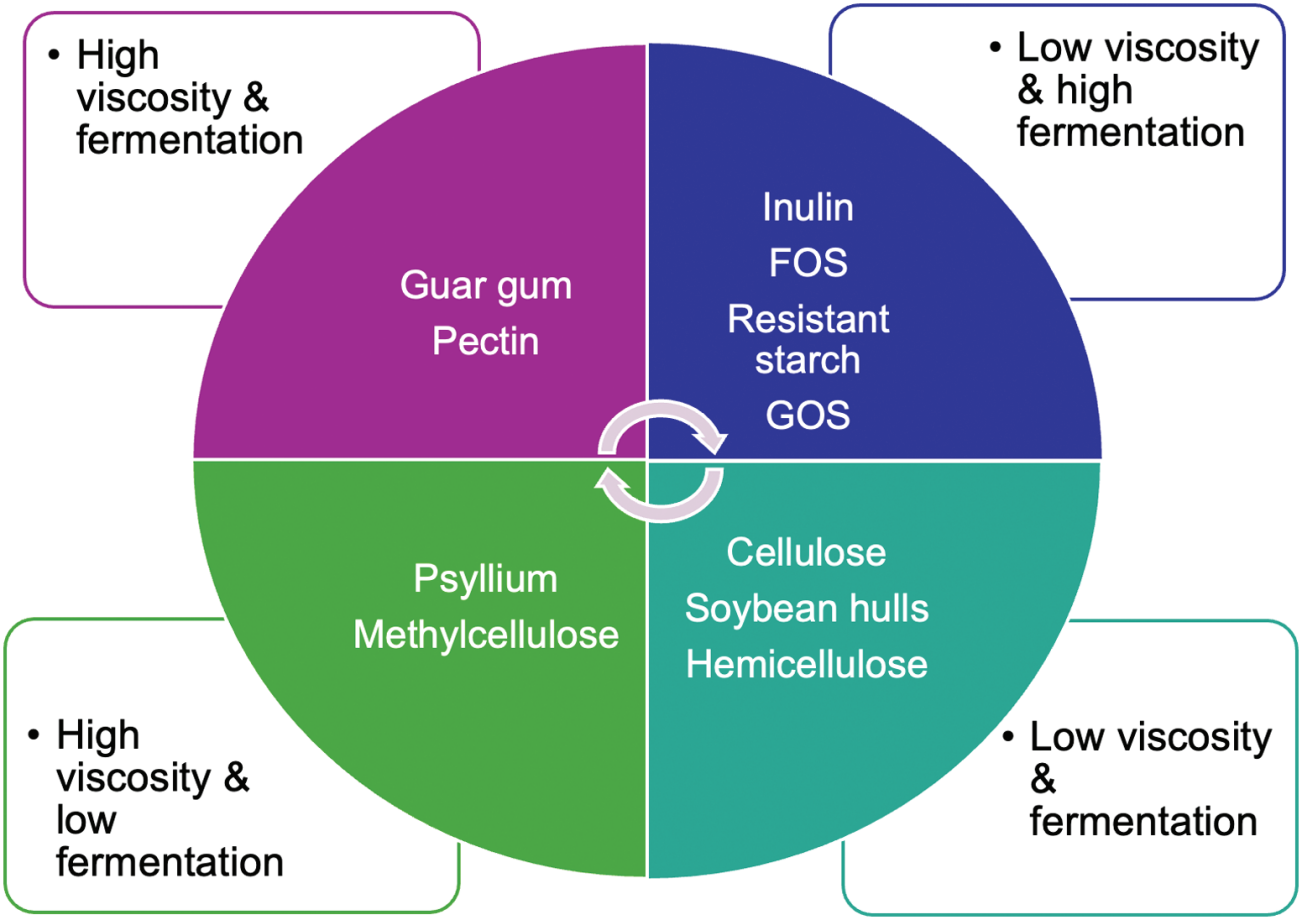
Fiber characterization based on their viscosity and fermentability profiles.

Absorbable CHO comprises monosaccharides (glucose, fructose, and galactose), which can be absorbed in the small intestine, whereas the digestible CHO includes disaccharides (such as sucrose and maltose) and polysaccharides (such as starch), which can be broken down to monosaccharides through host enzymatic action, absorbed, and further utilized on metabolic pathways to result in energy. On the other hand, indigestible CHO, given the absence of host digestive enzymes, are divided in two main groups: fermentable (such as soluble fibers, resistant starch, and some hemicellulose) and non/poorly fermentable CHO (such as cellulose and lignin; Adebowale et al., 2019; Kaushik et al., 2022; Figure 2). In the last few decades, indigestible CHO as dietary fibers have attracted the interest of food scientists and technologists due to several physiological and food processing benefits (Mudgil and Barak, 2013), which will be further discussed in this review.

## Nutritional Requirements

**Figure 3.**
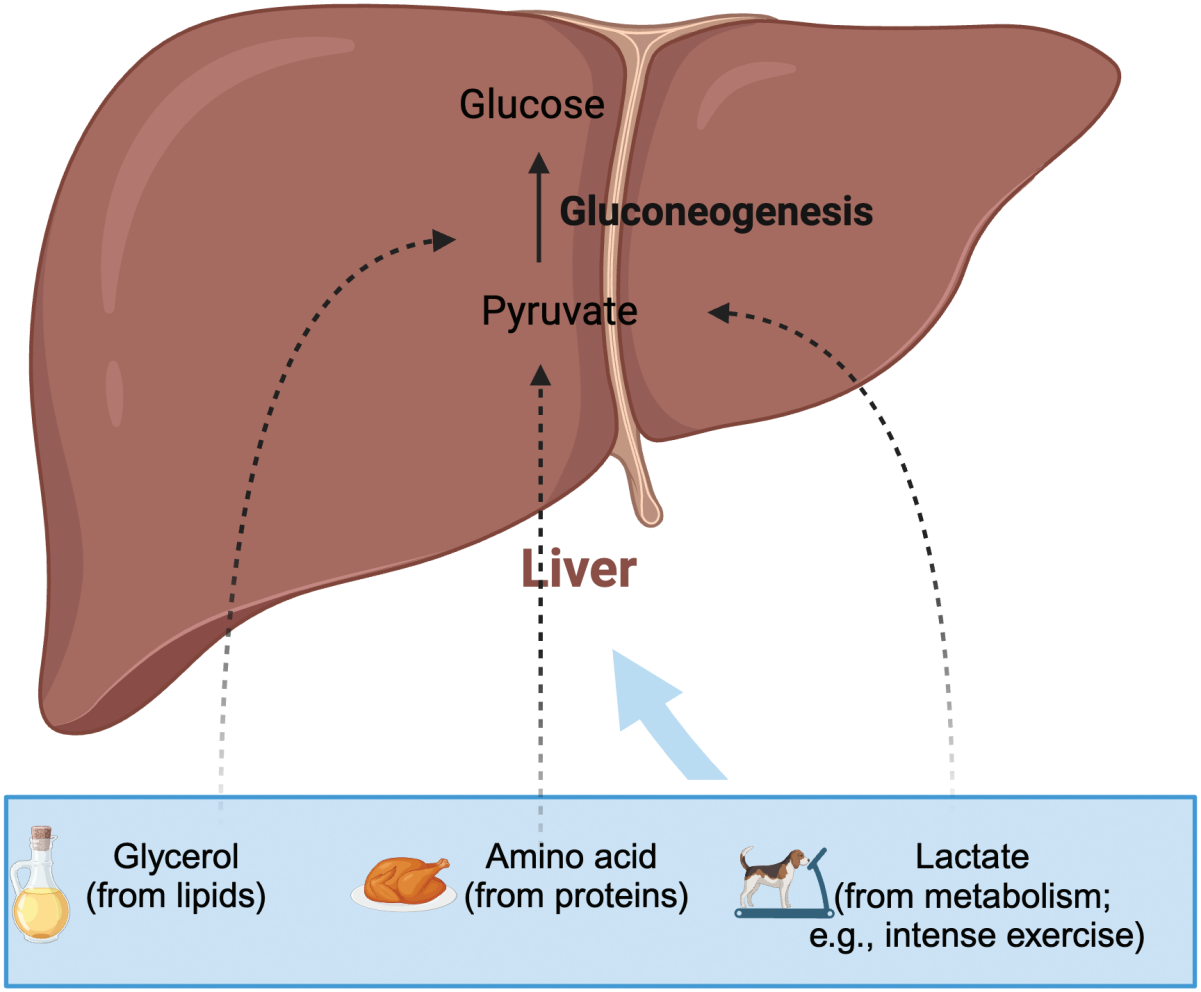
Biosynthesis of glucose from non-carbohydrate substrates.

Dogs and cats do not have a dietary requirement for CHO. They have metabolic glucose requirements instead. As in other species, specific tissues, such as the brain and specific cell types, such as red blood cells, rely on glucose for energy needs. It is therefore critical for the body to maintain a glucose supply for these tissues by strictly regulating the blood glucose concentration in the range of 3.9 to 6.7 mmol/L (70 to 120 mg/ dl) in cats, and 3.3 to 6.2 mmol/L (60 to 110 mg/dl) in dogs (Verbrugghe and Hesta, 2017; Idowu and Heading, 2018). CHO provide a valuable source of glucose in pet foods; however, when CHO are provided in insufficient amounts, the glucose can be metabolically supplied by gluconeogenic pathways (Nelson and Cox, 2008; Figure 3).

Certain life stages, such as gestation and lactation, require increased energy level; therefore, providing a diet with low CHO would force the activation of gluconeogenic metabolic pathways utilizing essential nutrients such as amino acids for glucose production, which may result in increased mortality of puppies, hypoglycemia, and acetonemia (Romsos et al., 1981; Kienzle et al., 1989). Romsos and colleagues (1981) compared the reproductive performance of dogs fed diets containing either 0% or 40% CHO. They observed that females consuming the diet with no CHO experienced severely reduced survival rate of their puppies (only one-third of the pups whelped survived for 3 days). Plasma glucose concentrations were similar in females fed the two diets, except in the week before whelping, when the ones fed the diet containing 0% of the metabolizable energy from CHO declined to concentrations of 15-20 mg/dl, when the reference range of blood glucose is between 60 and 120 mg/dl (Idowu and Heading, 2018). The hypoglycemic state of the females in that study were a result of unmatched demands for glucose that are increased during gestation for fetal development, resulting in low survival rates of the puppies.

Suckling puppies and kittens should not be given milk substitutes containing starch, given their lack of pancreatic amylase (Kienzle et al., 1989). In regards to the enzyme lactase, adult dogs have limited activity (3.3 U g^−1^ protein) compared with puppies (96 U g^−1^ protein; Kienzle, 1993). For this reason, lactose content in the diet should be limited for adult dogs, while puppies can digest, absorb, and metabolize lactose (Kienzle et al., 1989; Kienzle, 1993). Cats possess only a small capacity for starch digestion by endogenous intestinal enzymes, since the activities of disaccharidases in the small intestinal mucosa are not affected by the diet (Kienzle, 1993; Verbrugghe and Hesta, 2017). Consequently, consumption of excessive amounts of digestible CHO (> 5g/kg BW; Kienzle, 1993) will not only lead to increased glycemia, but will provide substrate for microbial fermentation in the colon, causing adverse gastrointestinal effects (Verbrugghe and Hesta, 2017). Additionally, processing improves the digestibility of starch in cat foods, because it promotes depolymerization, decreases molecular weight and improves enzymatic hydrolysis (Morris et al., 1977). Cooked corn starch had an apparent prececal digestibility (72.3 ± 16.7%) greater than that of raw corn starch (46.4 ± 36.3%; Kienzle, 1993).

Considering that different CHO have varying physiological effects, its recommended content in diets depends on amount of food consumed, caloric density of the food, and energy requirement of the animal (Legrand-Defretin, 1994). Although safe upper limits of selected CHO for adult dogs and cat maintenance diets have been suggested (i.e., sucrose 350 g/kg diet for dogs and 50-150 g/kg diet for cats on DM basis; lactose 100 g/kg diet for dogs and 50 g/kg diet for cats on DM basis; wheat bran 128 g/kg diet for dogs and 100 g/kg diet for cats on DM basis; NRC, 2006), there are no minimum requirements established.

## Digestible Carbohydrates (Starch)

Historically, cereal grains such as corn, wheat, and rice have been included in commercial companion animal diets as primary sources of complex CHO, specifically starch. Recently, products with “grain-free” formulas have become popular in the pet food market. However, it is important to keep in mind that “grain free” is not synonymous with “carbohydrate free”, and many of these products still contain alternative starch sources such as legumes and root vegetables and tubes (e.g., cassava, sweet potato, and potato). Consequently, grain-free diets may contain similar or greater starch content than pet foods containing cereal grains.

In addition to supplying the animal with a dietary source of glucose, starch plays an important role in the manufacturing of commercial pet foods. The majority of these products are produced using extrusion for dry and semi-moist products, or retort for wet products. Both processes provide the ideal conditions of moisture and heat to elicit the gelatinization of starch granules. During thermal processing, swelling and structural changes of the starch increase expansion and enhance the binding properties of the food matrix (Riaz and Rokey, 2012). Inclusion levels of starch can vary depending on the formula, with “low carbohydrate” diets having little to no starch, while diets with reduced fat content may include levels of starch as high as 50% (Spears and Fahey, 2004). Both extremes present challenges to processing considerations with low starch products having poor durability and formulas with starch inclusion greater than 65% resulting in extrudate that is too sticky and negatively impacts processing flow (Riaz and Rokey, 2012). Studies evaluating extruded pet foods have identified, moisture content, processing temperature, and starch source as factors that greatly influence the degree of gelatinization in these products and, subsequently, final product characteristics (Murray et al., 2001; Lankhorst et al., 2007; Pezzali and Aldrich, 2019; Alvarenga and Aldrich, 2020; Perry et al., 2022). Other studies have also reported on how inclusion of different CHO sources affect processing conditions during extrusion (Reilly et al., 2021; Traughber et al., 2021; Dainton et al., 2022).

Nutritionally, the degree of gelatinization is important because of its impact on starch digestibility. Structural and molecular properties such as granule size and crystallinity of starch from different ingredient sources can vary greatly and influence digestion kinetics (Martens et al., 2018). Physical barriers, such as plant cell walls or protein matrices, may block the access of digestive enzymes. However, gelatinization of the starch can disrupt these obstructions and help to overcome the inhibition in enzymatic action (Dhital et al., 2017). One study evaluating the concentrations of digestible starch fractions using an in vitro canine model reported that both low (79 to 93°C) and high (124 to 140°C) temperature extrusion increased the rapidly digestible starch content of barley, corn, rice, potato, sorghum, and wheat (Murray et al., 2001). Others have reported similar results in legumes, such as faba and kidney beans (Alonso et al., 2000). Starches that escape digestion and are not absorbed are referred to as resistant starches, and will pass on to the lower digestive tract where they act as dietary fibers (Spears and Fahey, 2004). The level of resistant starch reaching the colon is an important consideration as it stimulates bacterial fermentation, with greater concentrations having a negative impact on fecal quality (Goudez et al., 2011). In contrast, resistant starch can also beneficially modulate gut microbiota and fecal metabolites in pet animals (Jackson et al., 2020; Beloshapka et al., 2021). With the growing popularity of pet food products that are labeled as “raw” or “minimally processed”, there is a need to determine how these processing methods influence starch gelatinization and digestibility to avoid potential gastrointestinal intolerance.

## Dietary Fibers

Although not essential, dietary fiber sources have been included in formulations for canine and feline species due to their various physicochemical properties and ability to elicit different physiological responses and beneficial effects on health. With the knowledge of health benefits related to dietary fiber consumption in humans becoming more widespread, interest in applying these principals to pet species has grown. Efforts are being made by nutritionists and formulators to shift consumers’ perspective on fibrous ingredients from being “fillers” to functional components of the diet.

The FDA defines “fiber” as “non-digestible soluble and insoluble CHO (with 3 or more monomeric units), and lignin that are intrinsic and intact in plants; isolated or synthetic non-digestible CHO (with 3 or more monomeric units)” determined by FDA to have “physiological effects that are beneficial to human health” (FDA, 2016). These health benefits include reducing blood cholesterol, decreasing post-prandial blood glucose, aiding laxation, reducing blood pressure, increasing satiety relating to reduced energy intake, and increasing mineral absorption (FDA, 2016). There is still great variation among the compounds that meet these defining criteria and are considered dietary fibers (Table 1). Several categorical methods can be used when describing fiber including source (i.e., animal, plant, fungal, and chemically synthesized) and chemical structure (i.e., nondigestible oligosaccharides, non-starch polysaccharides, and resistant starch). They can also be categorized by certain characteristics that help to determine their functionality and mechanisms of action. These characteristics can be interrelated, and commonly include viscosity, fermentability, and solubility.

**Table 1.** Currently accepted isolated or synthetic non-digestible CHO and proposed non-digestible CHO to be added to FDA dietary fiber definition

Accepted isolated or synthetic non-digestible CHO	Proposed non-digestible CHO
• Beta-glucan soluble fiber • Psyllium husk • Cellulose • Guar gum • Pectin • Locust bean gum • Hydroxypropylmethylcellulose	• Mixed plant cell wall fibers • Arabinoxylan • Alginate • Inulin and inulin-type fructans • High amylose starch (resistant starch 2) • Galactooligosaccharide • Polydextrose • Resistant maltodextrin/dextrin • Cross linked phosphorylated resistant starch type 4 • Glucomannan • Acacia (gum arabic)

Viscosity describes the ability of a fiber to thicken and form a gel when hydrated. Viscous fibers have been associated with modulation of several physiological responses including gastric emptying and transit time as well as modulating glycemic response and circulating blood lipids (Dikeman and Fahey, 2006). Fermentability describes the degree of anaerobic digestion that can be performed by microbes in the digestive tract on the fiber substrate. Fibers are typically designated as non-fermentable, partially fermentable, or completely fermentable. Fermentation of dietary fiber has numerous health implications related to the modulation of the gut microbiota as well as production of fermentative by-products (Williams et al., 2017).

Solubility describes the ability of a fiber to dissolve in water or remain as distinct insoluble particles. In the literature, this method of distinguishing fiber types has been used to elucidate the differences in physiological response that were observed among different fiber treatments (Kimmel et al., 2000; Mudgil, 2017). However, a combination of these properties is likely to contribute to the physiological responses as soluble fibers are generally also characterized as viscous and fermentable while insoluble fibers are generally characterized as non-viscous and non-fermentable with a few exceptions (Elleuch et al., 2011).

While some traditional fiber sources, such as purified cellulose, may be very uniform in composition, most fibrous ingredients used in pet foods generally consist of a unique and diverse fiber profile that results in a combination of these characteristics. For example, beet pulp, considered to be one of the gold standard fiber sources in the pet food industry, has a fiber profile that includes individual fiber types with both reduced levels of fermentability, solubility, and viscosity such as cellulose, as well as pectin a highly viscous and fermentable fiber (Fahey et al., 1990; de Godoy et al., 2013). Recently, researchers have worked to evaluate a vast array of novel fiber ingredients, including avocado meal, soybean hulls, miscanthus grass, coconut fiber, chicory, citrus pulp, and orange fiber, to name a select few (de Godoy et al., 2015; Detweiler et al., 2019; Finet et al., 2021; Pacheco et al., 2021; Dainton et al., 2022). The main goal of those research studies was to identify fiber-rich ingredients that are economical, environmentally advantageous, and may otherwise contribute to food waste in the current food system, while describing the potential functionality of these fiber sources and blends in canine and feline nutrition and health.

The great diversity in fiber types and properties allows them to provide a variety of functions in the pet food industry. The use of fiber in modulating product density and acting as a carrier for minor ingredients such as vitamin and mineral premixes allow them to be functional components of the processing and manufacturing of pet food products (Donadelli et al., 2021). Additionally, the water binding properties of hydrocolloid fibers such as gums and pectin are essential in the texture development of wet food products such as pâté-style or chunks in gravy products (Dainton et al., 2021). While these processing attributes are of growing interest to pet food formulators and manufacturers, there is also great potential for fiber to act as a functional ingredient to the animal.

The goal of a functional ingredient is to provide the animal with a health benefit beyond basic nutrition (Kruger and Mann, 2003). Research in humans has worked to elucidate the physiological effects that fiber consumption has on metabolic action and gastrointestinal health as well as the role of prebiotic fibers in the maintenance of several biological systems (Carlson et al., 2018). While less data are available for companion animals, researchers have begun to evaluate if the same benefits can be observed in pet species.

Evaluating the relationship between dietary fiber consumption and attenuating symptoms of metabolic disorder has become increasingly important as the incidence of pet obesity rises. Several studies have reported the benefits of fiber in promoting glycemic control in dogs by slowing the movement of digesta through the gastrointestinal tract as well as the rate of pancreatic digestion and subsequently nutrient absorption (Nguyen et al., 1998; Graham et al., 2002; Muller et al., 2018; Rankovic et al., 2020). However, less is known regarding these effects in cats, with mixed results reported in the literature (Nelson et al., 2000; Bennett et al., 2006). Increasing fiber content, especially insoluble fibers, is a common strategy used to decrease caloric density without impacting intake volume in diets focused on promoting weight loss. While some research has hypothesized that fiber may help to promote satiety and further aid in weight management, the results in pet species are varied, and seem to depend on the composition of other macronutrients (e.g., protein and fat) in the test diets, warranting further evaluation in future studies (Butterwick et al., 1994; Fekete et al., 2001; Jewell et al., 2006; Weber et al., 2007).

**Figure 4.**
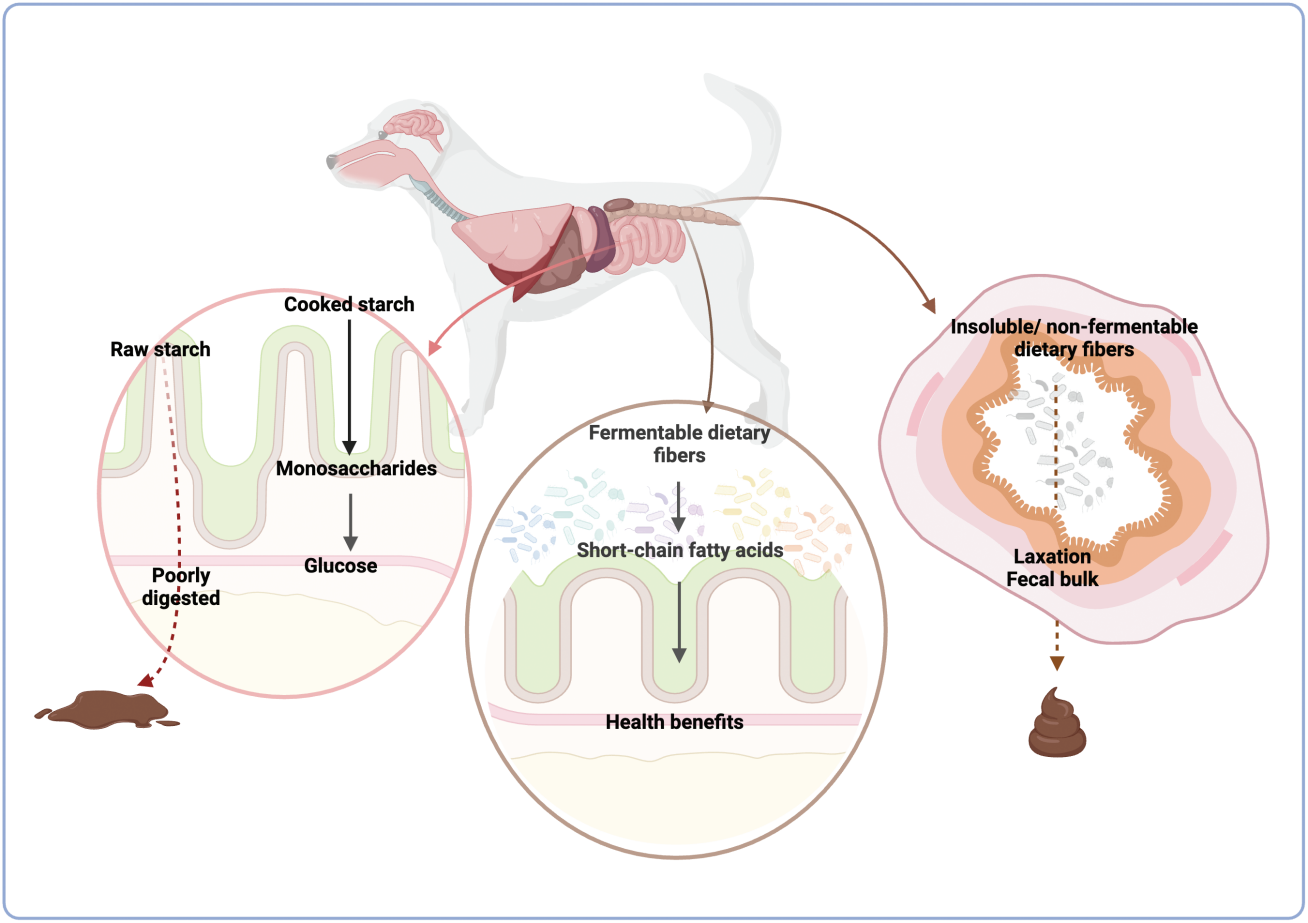
Overview of carbohydrate digestion and main physiological outcomes.

The role of fiber in maintaining gastrointestinal health and treating symptoms of intestinal distress has been more extensively studied. Fiber intake is known to aid laxation and promote ideal stool quality through several mechanisms. Both soluble and insoluble fibers contribute to fecal bulk and consistency. Insoluble fibers that resist fermentation contribute to fecal dry matter bulk, while soluble fibers bind water helping to increase fecal weight and soften stools. Increasing fecal bulk and weight helps to maintain regular elimination frequency (Fahey et al., 1990; Moreno et al., 2022; Figure 4).

## Prebiotics

The essential role of the gut microbiota on health has generated tremendous interest in modulating its composition and metabolic function. One of these strategies is the use of prebiotics, which have been defined as a “substrate that is selectively utilized by host microorganisms conferring a health benefit” (Gibson et al., 2017). Furthermore, prebiotics have the potential to affect nutrient digestibility, enhance immune function, and protect against disease by altering the gut microbiota and metabolites (Pawar et al., 2017).

The classification of a substance as a prebiotic for the gut relies on three criteria: 1) resist gastric activity, hydrolysis by mammalian enzymes and gastrointestinal absorption; 2) be fermented by intestinal microbiota; and 3) generate reproducible randomized controlled studies stablishing direct links between the prebiotic and health in the specific target host (Gibson et al., 2017; Scott et al., 2020). Prebiotics are frequently equated with dietary fibers, however, only a subset of dietary fibers qualify as prebiotics (Table 2). In fact, prebiotics may also derive from non-fiber substances, such as lactulose (Gibson et al., 2017). While the list of current recognized prebiotics is limited, other dietary compounds still lack data confirming health benefits, therefore they are classified as candidate prebiotics (Table 2). These include fiber compounds [e.g., xylo-oligosaccharide (XOS), β-glucans, and isomalto-oligosaccharide] and non-fiber compounds (polyphenols and polyunsaturated fatty acids; Scott et al., 2020). Dietary prebiotics most extensively documented to have benefits are the ones derived from dietary fibers, such as non-digestible oligosaccharides fructans and galactans (Scott et al., 2020). Currently, there is growing interest in understanding species-specific health benefits conferred by prebiotics and their potential in modulating gut microbiota and local and(or) systemic health.

**Table 2.** List of confirmed prebiotics and candidate prebiotics, food sources, and health endpoints targeted^*^

Confirmed prebiotic	Food source content of specific prebiotic	Health endpoints targeted
*Fiber derived*		
Galacto-oligosaccharides (GOS)	β-GOS produced enzymatically from lactose	Overweight and obesity (Gibson et al., 2017)
Fructo-oligosaccharides (FOS)	Asparagus (5%), leeks (11.7%), garlic (17.5%)	Satiety, overweight and obesity (Cani et al., 2009)
Inulin	Chicory (64.4%), onion (8.6%), Jerusalem artichoke (31.5%)	Constipation, overweight and obesity (Dewulf et al., 2013; Christodoulides et al., 2016)
*Non-fiber-derived*		
Lactulose	Synthetic disaccharide	Constipation (Ruszkowski and Witkowski, 2019)
Candidate prebiotics		
*Fiber derived*		
Resistant starch	Multiple food sources (corn, potato, tapioca)	Obesity (Snelson et al., 2019)
Polydextrose	Synthetic fiber	Infections and vaccine response (Valdez et al., 2014)
Xylo-oligosaccharide (XOS)	Wheat bran	Reduction of blood cholesterol (Palaniappan et al., 2021)
Isomalto-oligosaccharide (IMO)	Honey, sugar cane juice, sucrose	Constipation (Lan et al., 2020)
Β-Glucan	Soluble fiber found in oats and barley cereals (3-6%)	Obesity (Ferreira et al., 2022)
*Non-fiber derived*		
Polyphenolics	Berries, spices, nuts, seeds	Oxidative stress reduction (Alves-Santos et al., 2020)
Polyunsaturated fatty acids	Nuts, sunflower seeds, flax seed, salmon	Reduction of blood cholesterol (Kjølbæk et al., 2020)

^*^Adapted from Scott et al. (2020).

## Gut Health and Microbiota

### Fermentation and metabolic end-products

Gut microbes interact with host physiology on several levels. This can occur through direct contact with gut epithelial cells, which influences the development and maintenance of the host immune system, or through interaction with microbial-derived metabolites (Tizard and Jones, 2018; Pilla and Suchodolski, 2020). Microbes produce a variety of compounds including proteins, vitamins, gases, volatile fatty acids, and secondary bile acids. Some of these compounds such as proteins and vitamins are not able to be broken down or absorbed by the host in this region of the digestive tract and are excreted, if not utilized by gut bacteria. However, other compounds such as short-chain fatty acids (SCFA) can be absorbed by the colonocytes and utilized by host metabolism (Koh et al., 2016).

In the absence of oxygen, microbes utilize fermentation to breakdown carbon containing molecules such as CHO and protein to provide energy for growth and reproduction. Without oxygen, the fermentation substrates cannot be completely oxidized, resulting in end-products that retain some energy potential. Different microbes have distinct metabolic machinery, allowing them to utilize a certain subset of substrates and metabolic pathways to harvest energy and produce end-products (Pilla and Suchodolski, 2020). Since dietary fiber is the primary substrate that remains undigested by the host and reaches the large intestine, most metabolites produced are of saccharolytic fermentation. These include SCFA (i.e., acetate, propionate, butyrate), and gases (e.g., carbon dioxide and methane). Lactate and succinate are also produced, but are utilized quickly by other microbes as intermediates in the production of SCFA (Koh et al., 2016).

The production of SCFA has been cited as one of the primary benefits of fiber consumption, and the role of these compounds in maintaining host health has been extensively studied in the past decade. Once produced by the microbes, SCFA are absorbed into the gut epithelium. The majority of this happens via passive diffusion, but can also be carrier or transporter mediated (Dalile et al., 2019). Once absorbed, butyrate is the preferentially utilized by colonocytes as an energy source, and most remains in the colonic mucosa. Propionate and acetate can enter portal circulation before uptake and metabolism in the liver or extrahepatic tissues. The primary fate of acetate in the liver is fatty acid synthesis, while propionate acts as a gluconeogenic precursor (Koh et al., 2016). These compounds can also act as signaling molecules for several other biological systems in the host. For example, acetate and propionate are sensed by receptors that release peptide tyrosine tyrosine which promotes satiety. Butyrate acts as an inhibitor of histone deacetylase to suppress transcription and differentiation of various immune cells, ultimately having an anti-inflammatory effect which has an important role in maintaining the balance between immune response to pathogenic bacteria in the gut and tolerance of commensal bacterial species (Tizard and Jones, 2018; Dalile et al., 2019). Promoting enteric health and motility, suppressing tumor growth, and central nervous system signaling are also cited as critical functions of SCFA (Koh et al., 2016; Dalile et al., 2019; Caetano-Silva et al., 2023).

Recent advances in DNA sequencing technology and computational biology have revolutionized the field of microbiome, permitting the evaluation of the relationship between diet and the gut microbial population. Previous review articles have explored the effects of diets on gut microbiota of dogs and cats (Pilla and Suchodolski, 2021; Butowski et al., 2022), additional recent studies have evaluated the effects of dietary fibers, fiber blends, whole grains (Nogueira et al., 2019; Beloshapka et al., 2021; de Brito et al., 2021; Traughber et al., 2021; Finet et al., 2022; Palmqvist et al., 2023); or prebiotics (Panasevich et al., 2021) on gut microbiota of adult healthy dogs. In cats, previous reviews have described the effects of age, gastrointestinal disease, environment, and diet on the feline gut microbiome (Rochus et al., 2014; Pilla and Suchodolski, 2021). Since then, a few studies have been published evaluating the effects of carbohydrate and dietary fiber sources and their inclusion levels on fecal microbiota of adult healthy cats (Jackson et al., 2020; Finet et al., 2021; von Schaumburg et al., 2021; Lee et al., 2022).

## Conclusions

Although dogs and cats do not have defined nutritional CHO requirements, they provide a valuable source of energy in addition to supporting digestive function and overall health. Besides yielding a source of glucose to the diet, CHO also plays a vital role in manufacturing commercial pet foods. Considering that most of these products are produced using extrusion for dry and semi-moist products, or retort for wet products, both processes beneficiate from the gelatinization of starch granules. Moreover, non-digestible CHOs represent a segment of growing importance in companion animal nutrition since dietary fiber is closely related to gut health. Dietary fibers exhibit a diverse range of physicochemical properties and corresponding physiological effects. Characteristics such as solubility, fermentability, and viscosity are important determinants of the impact of fiber in the body. Further research is necessary to determine the effects of food processing and optimal inclusion levels of these fibers targeting the physiological states of dogs and cats. Advances in DNA sequencing and computational technology allowed the beginning of understanding interactions of nutrient-host-microbiome; however, many important questions remain unanswered.

## About the Authors

**Dr. Emanuela Kayser** has recently graduated with a doctoral degree from the Division of Nutritional Sciences at University of Illinois. Her research focused on functional ingredients such as probiotics, prebiotics, yeast products, and inositol effects in weight management, fecal microbiota, insulin sensitivity, oxidative stress, inflammatory biomarkers, and gene expression of adult dogs. She enjoys practicing sports and spending time with her husband, friends, and dogs.

**Shannon E. Finet**, while pursuing her bachelor’s degree in Animal Sciences, Shannon was introduced to nutrition research working as an undergraduate research assistant in the companion animal nutrition laboratory. During that time, she also pursued her interest in exotic species while working as a zookeeper at Scovill Zoo in Decatur, IL. After graduating, she combined these interests as a professional intern at the Animal Nutrition Center at Disney’s Animal Kingdom. She returned to the University of Illinois and received her master’s degree with a research focus on novel fiber blends in feline nutrition, and her PhD under Dr. Maria R. C. de Godoy with a research focus on evaluating traditional and novel fiber sources and their effects on gastrointestinal health and microbiota in canines.

**Dr. Maria R. C. de Godoy** is an associate professor and was a recipient of the 2021 Campus Distinguished Promotion Award at the University of Illinois. The focus of her research program is on companion animal and comparative nutrition with 3 main overarching goals: 1. analytical methods and animal models for evaluation of nutritional adequacy of novel ingredients; 2. the effects of processing on pet food quality, nutritional value, and safety; and 3. the role of nutraceuticals in health and wellness of pets. She has an internationally recognized research and teaching program. She has received numerous awards for her excellence in teaching and research. Dr. Godoy’s academic program showcases her innovative approach to science that further advances the pet food industry and the nutritional sciences community. **Corresponding author:**mgodoy2@illinois.edu
